# Video head impulse test in stroke: a review of published studies

**DOI:** 10.3389/fneur.2024.1339039

**Published:** 2024-03-01

**Authors:** Niranjana Jaganathan, Mohd Hazmi Mohamed, Ahmad Luqman Md Pauzi, Hasni Mahayidin, Ahmad Firdaus Hanapai, Wan Aliaa Wan Sulaiman, Hamidon Basri, Liyana Inche Mat

**Affiliations:** ^1^Department of Neurology, Faculty of Medicine and Health Sciences, University Putra Malaysia, Serdang, Selangor, Malaysia; ^2^Department of Otorhinolaryngology, Faculty of Medicine and Health Sciences, University Putra Malaysia, Serdang, Selangor, Malaysia; ^3^Department of Emergency, Faculty of Medicine and Health Sciences, University Putra Malaysia, Serdang, Selangor, Malaysia; ^4^Department of Pathology, Faculty of Medicine and Health Sciences, University Putra Malaysia, Serdang, Selangor, Malaysia

**Keywords:** video head impulse test, posterior circulation stroke, acute vestibular syndrome, vestibular neuritis, vestibulo-ocular reflex

## Abstract

Accurate and timely diagnosis of posterior circulation stroke in patients with acute dizziness is a challenge that can lead to misdiagnosis and significant harm. The present review sought to identify and describe published research on the clinical application of vHIT in posterior circulation stroke. vHIT, a portable device, has gained prominence in evaluating peripheral vestibular disorders and offers potential applications in diagnosing neurological disorders, particularly posterior circulation stroke. Several studies have shown that vHIT can differentiate between stroke and vestibular neuritis based on VOR gain values, with high sensitivity and specificity. The manuscript also discusses vHIT’s performance in differentiating between types of posterior circulation stroke, such as PICA, AICA, and SCA strokes. While vHIT has demonstrated promise, the review emphasizes the need for further research to validate its use as a tool to rule out stroke in acute dizziness patients in the emergency department. In conclusion, the manuscript underscores the potential of vHIT as a valuable addition to the diagnostic arsenal for acute dizziness, particularly in the context of posterior circulation stroke. It calls for further research and wider adoption of vHIT in clinical settings to improve patient care and reduce unnecessary costs associated with misdiagnoses.

## Introduction

Acute vestibular syndrome (AVS) is characterized by the abrupt onset of vertigo or dizziness, nausea or vomiting, intolerance to head motion, and an unsteady feeling. Vascular vertigo or dizziness should be considered in patients who show signs of AVS. According to the latest guideline published by the Committee for the Classification of Vestibular Disorders of the Barany Society, the following conditions can result in vascular vertigo/dizziness: stroke, transient ischemic attack (TIA), isolated labyrinthine infarction/hemorrhage, and vertebral artery compression syndrome (VACS) (1). The difficulties of distinguishing peripheral vestibular disorders such as vestibular neuritis (VN) vs. posterior circulation stroke (PCS) have always been a challenge to the frontliners, leading to frequent misdiagnosis and potentially serious harm to patients.

The video head impulse test (vHIT) provides a portable and objective method for distinguishing between these conditions. It utilizes head-mounted goggles equipped with a high-speed camera and sensors to evaluate head velocity (2). Worn by the patient, these goggles record the movement of their eyes while their head undergoes rapid rotations in various directions. This allows the clinician to assess the gain, latency, and symmetry of the vestibulo-ocular reflex (VOR), a crucial reflex enabling us to maintain a fixed gaze on a target while our head is in motion. In contrast to subjectively observing corrective saccades resulting from the VOR, vHIT offers an objective measurement of both overt and covert corrective saccades. Physiologically, vHIT works by measuring the VOR gain of each semi-circular canal by calculating the duration ratio between head impulse and gaze deviation (3). A normal VOR gain value is >−0.81 for horizontal canals and >−0.71 for vertical canals. Any value below the cut-off threshold is considered abnormal (4).

## Materials and methods

An electronic search was performed by searching the following databases: PubMed, Scopus and Google Scholar. Articles are searched using the following search terms: video head impulse test, video oculography, acute vestibular syndrome, posterior circulation stroke, acute vertigo, and acute dizziness. We only consider publications in the English language before 31 October 2023. Congress-related published abstracts were excluded. Reference lists of related publications were also examined for further sources not identified in online searches. This search strategy yielded 18 published works.

Thus, a total of 18 clinical studies were selected and their clinical data were summarized in Table 1.

**Table 1:** Summary of published studies of VHIT on stroke.

Author	Newman Toker et al. (46)	Mantokoudis et al. (48)	Mantokoudis et al. (7)	Guler et al. (8)	Calic et al. (6)	Lee SH et al. (18)	Lee JY et al. (45)	Machner et al. (38)	Nham B et al. (19)	Siepmann et al. (14)	Machner et al. (38)	Nam GS et al. (51)	Thomas JO et al. (41)	Morrison et al. (5)	Korda et al. (21)	Korda A et al. (39)	Lee SH et al. (52)	Ha SH et al. (50)
Year published	2013	2015	2016	2017	2020	2020	2020	2021	2021	2021	2021	2021	2022	2022	2022	2022	2023	2023
Journal	Stroke	Otology and Neurootology	Journal of Vestibular Research	Journal of Vestibular Research	Clinical Neurophysiology	Frontiers in Neurology	Journal of Clinical Neurology	European Journal of Neurology	Journal of Neurology	Journal of Clinical Medicine	Frontiers in Neurology	Frontiers in Neurology	BMJ Neurology	Journal of Neurology	Frontiers in Neurology	Frontiers in Neurology	Frontiers in Neurology	Frontiers in Neurology
Country	USA	USA	USA	Turkey	Australia	Korea	Korea	German	Australia	Germany	German	Korea	Australia	Switzerland	Switzerland	Switzerland	Korea ED	Korea
Location	ED	ED	ED	ED	ED and outpatient	ED	Dizziness Center	ED	ED and outpatient	ED	ED	ED	ED	ED	ED	ED		Stroke unit
Type	ICS impulse USB googles, Otometrics, Taastrup, Denmark	ICS impulse USB googles, Otometrics, Taastrup, Denmark	ICS impulse USB googles, Otometrics, Taastrup, Denmark	ICS Impulse, GN Otometrics, Taastrup, Denmark	ICS impulse USB googles, Otometrics, Taastrup, Denmark	ICS impulse USB googles, Otometrics, Taastrup, Denmark	Not provided	EyeSeeCam HIT System (Autronics, Hamburg, Germany)	ICS impulse USB googles, Otometrics, Taastrup, Denmark	EyeSeeCAm HIT, Interacoustics, Middlefart, Denmark	EyeSeeCam HIT System (Autronics, Hamburg, Germany)	SLMED, Seoul, Korea	ICS impulse USB googles, Otometrics, Taastrup, Denmark	EyeSeeCam, Munich	EyeSeeCam, Munich	EyeSeeCam (EyeSeeTec GmbH)	ICS impulse USB googles, Otometrics, Taastrup, Denmark	SLMED, Seoul, South Korea for VN patients; GN Otometrics, Taastrup, Denmark
Operator	Neuro-otologist	Post-doctoral research fellow/trained nurse	Post-doctoral research fellow/trained nurse	Neuro-otology research fellow	Not provided	Neuro-otologist	Not provided	Medical-technical assistant	Neuro-otologist	Not provided	Medical-technical assistant	Experienced technician	Trained audiologists	Not provided	Neuro-otologist	Neuro-otologist	Neuro- otologist	Trained technician
Inclusion criteria	AVS (<7 days)	Continuous vertigo or dizziness lasting at least 1 h	AVS	AVS	AVS	Lateral medullary stroke	Dizziness	AVS	AVS	AVS (<7 days)	AVS	AVS	AVS	AVS	AVS	AVS	Isolated nodular stroke	Acute stroke (within 24 h)
Exclusion criteria	Previous vestibular or oculomotor disorder, new head trauma, acute drug/alcohol intoxication	Prior vestibular or oculomotor disorder	AICA stroke	Previous history of vestibular or oculomotor disorders	Unable to complete test due to severe vertigo	Lack of dizziness/vertigo symptoms	Data reliability	Onset symptoms >72 h, symptoms remitted less than 24 h, patient did not undergo MRI or vHIT, inflammatory diseases of CNS	Patients with a proven cardiac rhythm disturbance, orthostatic hypotension, or other non-vestibular cause such as anemia or hypoglycaemia	Postural vertigo, visual/cervical spine impairment, history of vestibular dysfunction	Patients who did not undergo vHIT	previous history of stroke or vestibular disorder, medullary or other brainstem or supratentorial stroke	Patients who did not undergo HIT, vHIT and MRI	Non-persistent symptoms	Patient <18yo, symptoms remitted less than 24 h, ED visit >72 h after symptom onset	Patient <18yo, symptoms remitted less than 24 h, ED visit >72 h after symptom onset	Existent opthalmoplegia	Concomitant lesions in anterior circulation, altered mental status and active systemic diseases
Time to vHIT	<7 days	<1 week	<10 days	Acute	<14 days	<16 days	<10 days	<24 h	<3 days	<24 days	<24 h	Not mentioned	Next business day	<3 days	Not mentioned	Not mentioned	<5 days	<2 days
Number of patients, *n*	12 patients (6 stroke, 6 peripheral vestibular)	26 patients (10 stroke)	23 AVS patients (5 stroke)	52 AVS patients (16 stroke)	22 stroke patients	17 stroke patients	893 patients (11 stroke)	38 AVS (14 stroke, 24 VN)	539 patients (46 stroke)	30 AVS patients (11 stroke)	171 AVS (37 stroke, 85 stroke)	17 PICA stroke, 17 VN	133 AVS (20 stroke)	152 AVS patients (27 stroke)	46 AVS (11 stroke, 35 AUVP)	57 AVS (18 stroke, 39 AUVP)	8 isolated nodular stroke	80 stroke
Study type	Prospective	Prospective	Prospective observational	Prospective Cross-sectional	Prospective	Prospective	Retrospective	Retrospective	Prospective cohort	Prospective	Retrospective	Prospective	Prospective	Prospective cross-sectional	Prospective	Prospective	Retrospective	Prospective
Bedside vestibular test	HINTS	HINTS	-	HINTS	-	HINTS	-	HINTS	Structured assessment	HINTS-plus	-	-	HINTS	Caloric test	HINTS	-	-	-
Imaging	MRI/CT	MRI	MRI	MRI	MRI	MRI	Not provided	MRI	MRI	MRI	MRI	MRI	MRI	MRI	MRI	MRI	MRI	MRI

AVS, acute vestibular syndrome; AUVP, acute unilateral vestibulopathy; ED, emergency department; HINTS, Head impulse, nystagmus and test of skew test; MRI, magnetic resonance imaging; vHIT, video head impulse test.

Due to the shortage of research on video head impulse tests in stroke, there was a lack of consistency in the outcome measures used and in the theoretical and methodological approaches employed. Furthermore, the gain measurements for the primary VOG device, primarily obtained using the EyeSeeCam and the Otometrics device, exhibit variations due the difference in measurement methods. Consequently, direct comparisons of the calculations are not feasible due to these differences.

## Results

All studies included in this review were published between 2013 and 2023. Regarding study design, there were 14 prospective and 4 retrospective studies. The sample size ranged from 12 to 893 participants. As expected, there was a smaller number of stroke patients compared to peripheral causes. Most studies use MRI scans as neuroimaging of choice, while 1 study uses both CT and MRI. 1 study did not provide this information.

As for the country of origin, 3 studies were conducted in the US, 5 in Korea, 3 in Australia, 3 in Switzerland, 3 in Germany, and 1 in Turkey. Various types of vHIT machines were used in the study, most used is the ICS impulse USB googles, Otometrics, Taastrup, Denmark while 2 studies used the EyeSeeCam, Munich and another used the EyeSeeCAm HIT (Interacoustics, Middlefart, Denmark). Another study from Korea did not provide the name of the machine used, but another 2 studies in this country used SLMED, South Korea. In terms of the operator for the machine, most were performed by neuro-otologists.

vHIT was conducted shortly after the onset of AVS symptoms in the majority of studies. AVS is defined as acute onset of continuous vertigo or dizziness lasting at least 1 h. In addition to AVS, our review encompassed investigations on vHIT involving isolated nodular stroke and lateral medullary stroke. Many studies opted to exclude individuals with prior vestibular or oculomotor disorders, recent head trauma, and acute drug/alcohol intoxication, as these conditions can elicit nystagmus. Furthermore, some studies ruled out participants with cervical impairment, given the infeasibility of performing vHIT on individuals with spine or neck injuries. Additionally, certain studies integrated bedside clinical assessments alongside vHIT or other tests evaluating vestibular function. vHIT was performed acutely after the onset of dizziness in most studies Apart from AVS, we also included studies of vHIT on isolated nodular stroke and lateral medullary stroke.

### Comparison of VHIT to bedside testing

HINTS constitutes a battery of bedside clinical tests, including head impulse test (HIT), assessment of nystagmus, and evaluation of skew deviation (22–24). HIT is a straightforward bedside clinical examination in which the clinician passively rotates a patient’s head abruptly while observing the VOR (3).

Despite its usefulness as a bedside tool in patients presenting with acute dizziness in the emergency department, the major drawback to HINT is its subjectivity, making it highly operator-dependent (16, 25, 26). Detecting overt corrective saccades requires experience, and an inexperienced operator may easily overlook these findings (7, 8, 27).

Hotson and Baloh (28) have shown that the presence of direction-changing horizontal nystagmus in any gaze direction consistently implies a central localization. Conversely, unidirectional horizontal nystagmus can happen in lesions that are peripheral or central (29). A significant decrease in the horizontal head impulse VOR gains can occur from unilateral or bilateral (positive HITs) lesions of the vestibular nucleus, flocculus, or nucleus prepositus hypoglossi (30–32). In such cases, it is imperative to assess the integrity of the horizontal VOR using vHIT to distinguish between central and peripheral localization. If the vHIT results are normal, it is evident that the lesion causing the unidirectional nystagmus spares the vestibular periphery, pointing toward a central lesion as the likely cause.

When skew deviation is detected during cover testing, a central lesion is likely present. A substantial skew deviation, as seen in AVS, is more frequently linked to acute stroke than minor skew deviations found by cover testing, which may occasionally appear in vestibular neuritis. Such a pronounced skew deviation should be regarded as a red flag, prompting the need for additional investigations (3, 33, 34). The combination of these three oculomotor findings has proven to be more sensitive (96.5%) and specific (84.4%) than MRI brain imaging for detecting posterior circulation stroke (13, 35).

HINTS examination has also been combined with other bedside examinations, such as the Dix-Hallpike test, STANDING, ABCD2 score and HINTS Plus to enhance stroke detection (11, 14, 36).

HINTS exam has demonstrated high sensitivity in detecting PCS (7, 8, 18, 37). However, vHIT is shown to be more specific in detecting PCS (16, 38). This is particularly useful in settings without neuro-otological experts who can perform the HINT bedside examination reliably. vHIT demonstrated an overall accuracy of 94.2% in detecting central pathology, boasting 100% sensitivity and 88.9% specificity. In contrast, experts evaluating bedside HINTS achieved slightly lower accuracy at 88.3%, comprising 90.9% sensitivity and 85.7% specificity (39).

Common neurological bedside examinations like NIHSS (National Institutes of Health Stroke Scale) scoring are also insensitive to detecting posterior circulation stroke, making it challenging for ED physicians to confidently rule out acute stroke in patients with acute dizziness (40).

Despite the high specificity and sensitivity of vHIT in detecting acute stroke, the importance of clinical assessment should not be undermined. The integration of subjective and objective evaluations of VOR gain, as seen in the combination of HINT and vHIT, has demonstrated an enhancement in distinguishing between PCS and VN (13, 41). Re-examining patients at the conclusion of the 24-h period is imperative, it should be noted, for the purpose of differentiating between vertigo and other conditions, such as migraine, Meniere’s disease, and TIA (1).

### vHIT vs. another non-invasive vestibular function test

#### Scleral search coil

Before the development of vHIT, the gold standard for measuring VOR gain was the scleral search coil (SSC). This technique involves a lightweight copper coil implanted into a circular-shaped silicon, which is later attached to the sclera (42). However, due to its invasive nature, it is impractical for clinical usage (43). Chen et al. examined the differences in HIT gains and compensatory saccades in PCS and VN using dual-search coils. In VN, there were asymmetric gain reductions and uneven compensatory saccades. In contrast, AICA strokes resulted in bilaterally reduced HITs, along with relatively small corrective saccades. Finally, PICA strokes demonstrated a directional bias, with HIT gains increased toward the opposite side of the lesion, accompanied by the smallest saccades overall (44). The emergence of vHIT as a quick, reliable, and validated tool for measuring VOR gain has led to its comparison with the SSC in several clinical studies (1, 10, 17).

#### Caloric test

vHIT has also advanced to replace the caloric test in assessing semi-circular canal function in vestibular patients (2, 5, 9, 12). However, caloric testing may be more relevant in conditions like Meniere’s disease and vestibular migraine (5).

In this test, patients lie supine at 30 degrees, and warm and cold-water solutions are irrigated into the affected ear within 25 to 30 s, with resulting nystagmus observed (37, 45). Compared to vHIT, bithermal caloric testing has lower sensitivity and specificity in detecting stroke in patients with acute dizziness. An abnormal caloric test and normal vHITs are associated with peripheral causes of dizziness (6). In cases of normal vHIT, it is advisable to proceed with caloric testing to rule out peripheral vertigo.

#### Vestibular-evoked myogenic potentials

Another vestibular function test is the Vestibular-Evoked Myogenic Potentials (VemPs), which consist of two major components: cVEMPs and oVEMPs. Asymmetry ratios (AR) are then used to detect unilateral otolith dysfunction. Although vEMPs have high specificity (90.9%) for detecting vestibular neuritis, especially in the absence or asymmetry of oVEMPs, they are less useful in detecting posterior circulation stroke. cVEMPs also show similar abnormal AR in both VN and PCS (19).

#### Subjective visual horizontal

Subjective Visual Horizontal (SVH) is performed by asking the patient to sit upright in a dark room, looking at an illuminated line presented at various angles from a 1.5 m distance. Contraversive SVH deviation, indicating lesions rostral to the pons, is a precise yet insensitive indicator of PCS. SVH is more likely to produce abnormal results in either VN or PCS, making it a poor discriminatory test to distinguish between the two (19, 37).

Several studies have incorporated the use of multiple tools to improve the detection of stroke in acute vestibular syndrome (9, 46). Structured history-taking and physical examination, including HINTS/HINTS PLUS, serve as the backbone in most of these studies. This is then followed by additional vestibular testing, such as vHIT, VEMP, and bithermal caloric testing. When combined, these tools have shown promising results in enhancing sensitivity and specificity.

### vHIT vs. neuroimaging

Regrettably, neuroimaging studies run the risk of overlooking PCS. The commonly employed investigation modality for stroke in emergency departments, brain computed tomography (CT), exhibits notably low sensitivity in identifying this condition. Retrospective studies suggest that CT may have as low as 42% sensitivity for ischemic stroke in cases of dizziness. However, a lack of awareness regarding CT’s limitations in assessing dizziness may contribute to its overuse and the potential for misdiagnosis. Additionally, even early MRI Brain with diffusion-weighted imaging may fail to detect up to 20% of acute PCS (47). The current gold standard for detecting PCS is an MRI of the brain performed more than 48 h after the onset of dizziness. In accordance with published studies, the majority employed MRI brain as a confirmatory test for PCS, with the exception of a 2013 study by Newman Toker et al., which utilized either CT or MRI brain. Although MRI is more commonly available nowadays, carrying out this procedure for every patient experiencing dizziness in a busy and overcrowded emergency department is not feasible and would consume a significant amount of time.

Patients generally tolerated vHIT examination well (7, 48). In the future, vHIT could be a valuable diagnostic tool for patients suspected of stroke who cannot undergo an MRI due to reasons such as claustrophobia or the presence of incompatible MRI devices on-site. It is also noteworthy that vHIT is considerably more cost-effective than an MRI machine, making it potentially useful in district hospitals or settings with limited resources.

### Findings in posterior circulation stroke

The utilization of vHIT for detecting PCS in cases of AVS has been established through various clinical studies (21, 47, 48). HIT gain and catch-up saccades characteristics can distinguish between PCS and VN (6, 19, 37, 46, 47). Mean VOR gain assessment achieves a 91% accuracy in differentiating PICA strokes from VN. PCS patients exhibited low or normal VOR gain, increased catch-up saccade amplitude, and saccade frequency compared to healthy age-matched controls (46). There is also significant refixation-saccade prevalence difference between PCS and VN. Additionally, in normal controls, the first and cumulative saccade amplitudes, initial saccade peak velocities, and duration were smaller, while in PCS, they were higher, and in VN, they were the highest. Utilizing artificial intelligence (AI) for the analysis of video Head Impulse Test (vHIT) data indicates a promising direction for future exploration (24).

#### PICA stroke

The territory most affected in PCS is the PICA (4, 15, 41, 49, 50). In a study by Mantokoudis et al., involving 26 patients with AVS and utilizing ICS impulse, a vHIT device, it was found that among the 7 patients diagnosed with PICA stroke, the mean VOR HIT gains fell within the normal range, with no discernible difference between ipsilesional and contralesional infarcts (21). Similar qualitative findings were observed, with over 99% of PICA strokes displaying normal clinical horizontal head impulse test results (18, 20, 51).

Another study by Guler et al. concluded that VOR gains in PICA-SCA stroke (pure cerebellar stroke with no brainstem involvement) were normal, showing no asymmetry. In AICA-PICA stroke, low VOR gain was observed on both ipsilesional and contralesional sides compared to healthy controls (8). Meanwhile, Calic et al. combined VOR gain and individual saccade parameters to enhance PCS detection, revealing that PCS is associated with normal or reduced VOR gain. Yet, the combined measures did not improve the differentiation between PCS and VN (19).

PICA stroke was found to be associated with preserved VOR gain and smaller corrective saccade amplitude in the ipsilesional horizontal canals (51, 52). While VOR gain proves useful in distinguishing between PCS and VN, there is no significant difference in VOR when comparing lesions in the midbrain, medulla, or pons of PCS (15). A small single-center study also concluded that preserved aVOR gain was found in patients with isolated heminodular stroke, which involves the nodulus with or without associated cerebellar structures supported by the medial posterior inferior cerebellar artery (mPICA) (52). The combination of normal VOR gain and absence of VOR asymmetry allowed investigators to correctly identify PCS in patients with AVS.

#### Lateral medullary stroke

The lateral medullary region of the brainstem is primarily supplied by PICA. A complete infarction in this area can lead to Wallenberg syndrome, characterized by acute vertigo, ipsilateral Horner’s syndrome, ataxia, and facial hypesthesia, along with contralateral hemisensory deficits. Examinations may also reveal associated nystagmus and skew deviation (53).

In a study conducted in a South Korean university hospital involving 17 patients with unilateral lateral medullary syndrome, the majority of patients (88%) exhibited normal aVOR gain. Only two patients demonstrated unilaterally reduced aVOR gains, both of which were mild and restricted to specific semicircular canals. The investigators attributed these findings to minimal or no involvement of the vestibular nuclei, as evidenced by MRI brain scans (51).

#### AICA stroke

More pronounced changes in VOR gain were observed in cases involving AICA strokes. Both vHIT studies demonstrated bilaterally reduced VOR gains without asymmetry in this specific subtype of PCS (8, 49). Guler et al. concluded that VOR gain is more likely to be impacted in AICA-PICA stroke (brainstem infarct) since the AICA supplies the vestibulo-oculomotor system and connecting oligosynaptic pathways (8). Similarly, in VN, VOR gains are significantly reduced bilaterally, potentially causing confusion with AICA strokes (5). False positive HIT examinations are more likely to be encountered in AICA infarction compared to PICA (19, 32, 54, 55).

#### SCA stroke

Similar to PICA stroke, VOR gain findings in SCA stroke can be normal or slightly diminished without asymmetry (7, 44).

## Limitations of vHIT

Despite the numerous benefits it offers, the vHIT has certain limitations. Factors like artifacts and technical issues, such as goggle slippage, can potentially affect vHIT results, emphasizing the need for a proficient operator to improve accuracy. Additionally, the vHIT machine relies on the operator’s skills and experience, making it operator-dependent and influencing the test’s overall quality. Moreover, vHIT might not be suitable for certain patients, particularly those with a prior history of neck or spine injuries. Financial considerations, encompassing both purchase and maintenance costs, pose additional constraints on vHIT. Furthermore, there is a notable learning curve essential for attaining proficiency in employing vHIT across diverse settings like the emergency department, wards, and outpatient clinic.

## Conclusion and perspective/general conclusions and suggestions for future research

The purpose of this review was to identify published studies that have used vHIT in detecting posterior circulation stroke. There has been growing interest among neurologists and researchers on the topic of vHIT in recent years. However, these studies involved a small number of patients, and most were conducted in a single-center setting. This may be attributed to the high cost of performing MRI and the lack of experienced personnel to conduct and interpret the vHIT machine.

While the vHIT does have some limitations, its many advantages make it a critical tool in the diagnosis and treatment of acute dizziness. In a busy emergency department, vHIT may be useful in triaging patients with acute dizziness to speed up the diagnosis of acute stroke. In addition, many acutely dizzy patients with peripheral vestibular causes for their symptoms are over-tested, misdiagnosed, and undertreated. The expenses associated with unwarranted imaging and hospital admissions for these patients can be substantial. Thus, accurate and efficient diagnosis for these patients will likely save lives and reduce costs through prompt and appropriate treatments.

Patients with vHIT findings that are suggestive of peripheral vestibulopathy may be triaged to the green zone or seen in outpatient settings. This will lessen the workload of emergency department staff and will in turn improve the hyperacute care of stroke patients. Similarly, patients who presented to district hospitals can be effectively triaged into those that need to be sent to tertiary hospitals to receive higher level stroke care or those that can be treated conservatively.

In summary, vHIT demonstrated encouraging indications as a diagnostic tool for identifying posterior circulation stroke in acute situations. Further research is essential to validate the efficacy of vHIT as a diagnostic test for ruling out stroke in patients with acute dizziness in the emergency department.

## Author contributions

NJ: Writing – original draft. MM: Data curation, Supervision, Writing – review & editing. AM: Resources, Writing – review & editing. HM: Supervision, Writing – review & editing. AH: Writing – original draft. WW: Conceptualization, Methodology, Supervision, Writing – review & editing. HB: Writing – review & editing. LI: Conceptualization, Funding acquisition, Writing – review & editing.
